# Supplementary material for: Consistent Biomarkers and Related Pathogenesis Underlying Asthma Revealed by Systems Biology Approach
Source: Int J Mol Sci. 2019 Aug 19;20(16):4037. doi: 10.3390/ijms20164037 (PMC6720652; doi:10.3390/ijms20164037)
Supplement: Supplementary file 1 [file ijms-20-04037-s001.zip › ijms-566768-supplementary-XML/Supplementary Material S2-Reports of Quality control for ten datasets.zip!/Supplementary Material S2-Reports of Quality control for ten datasets/QC_report_for_GSE4302/hm.pdf]

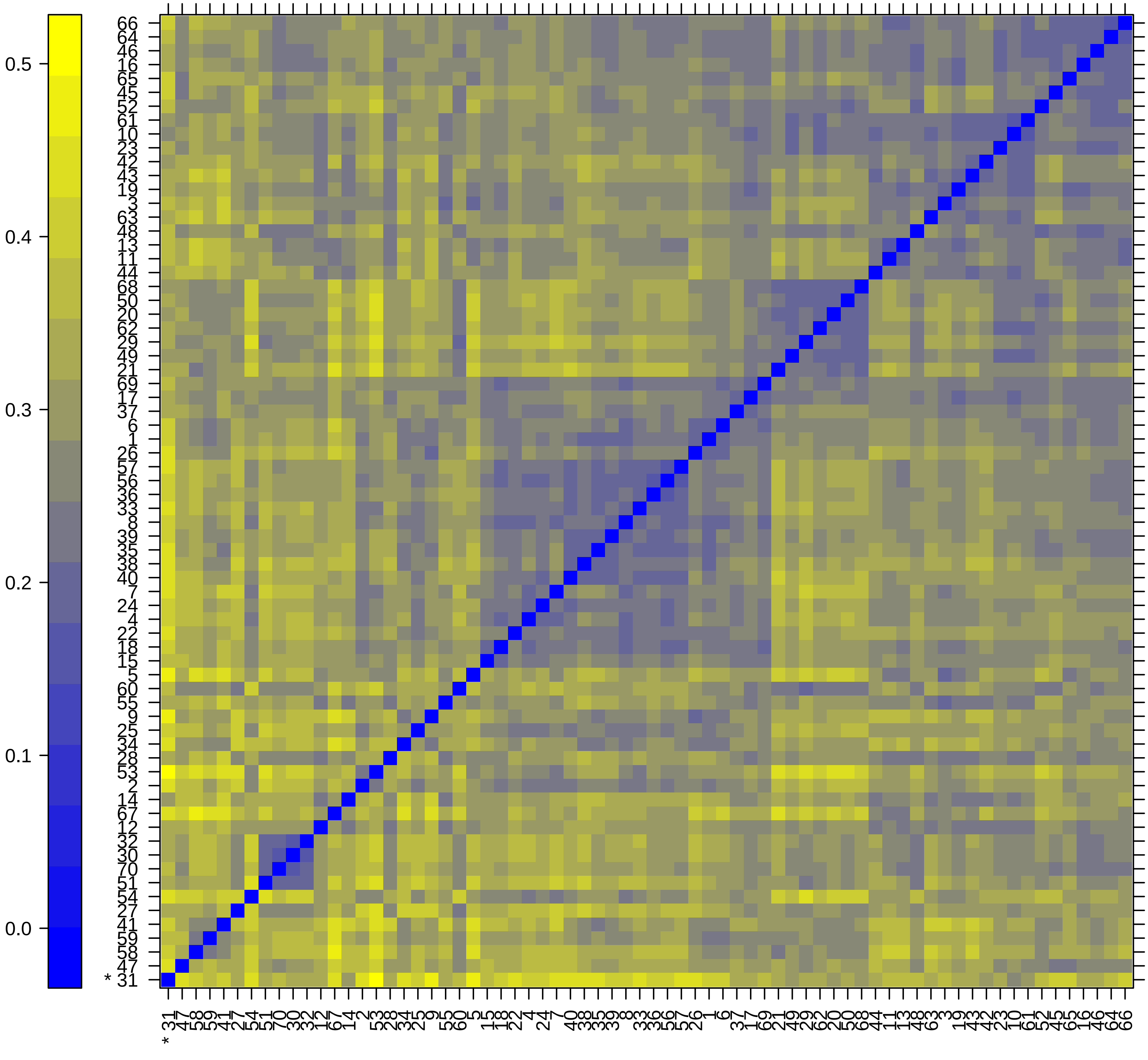
0.5
0.4
0.3
0.2
0.1
0.0
66 64 46 16 65 45 52 61 10 23 42 43 19 3 63 48 13 11 44 68 50 20 62 29 49 21 69 17 37 6 1 26 57 56 36 33 8 39 35 38 40 7 24 4 22 18 15 5 60 55 9 25 34 28 53 2 14 67 12 32 30 70 51 54 27 41 59 58 47 * 31
* 31 47 58 59 41 27 54 51 70 30 32 12 67 14 2 53 28 34 25 9 55 60 5 15 18 22 4 24 7 40 38 35 39 8 33 36 56 57 26 1 6 37 17 69 21 49 29 62 20 50 68 44 11 13 48 63 3 19 43 42 23 10 61 52 45 65 16 46 64 66
